# Simulation of Billet Shapes Using Multiple Atomizers

**DOI:** 10.3390/ma19204390

**Published:** 2026-10-19

**Authors:** Runfang Xiang, Xiyu Zhang, Fuyang Cao, Lunyong Zhang, Jianfei Sun, Hongxian Shen

**Affiliations:** School of Materials Science and Engineering, Harbin Institute of Technology, Harbin 150006, China; 23b909106@stu.hit.edu.cn (R.X.); zhangxiyuhit@163.com (X.Z.);

**Keywords:** spray forming, billet shape, numerical simulation, multiple atomizers

## Abstract

Large-scale segregation-free castings are in high demand for aerospace applications, yet the production of large-sized spray-formed billets remains poorly defined. This research addresses the preparation of such billets. To address the preparation of large-scale spray-formed Al–Zn–Mg–Cu aluminum alloys, numerical simulation methods were employed to investigate the billet fabrication process in spray forming, and the distribution characteristics of pores within the billet were analyzed. The results indicate that the drawing speed of the substrate is the primary factor that controls the billet dimensions. The slower the drawing speed, the larger the billet diameter. The eccentric distance and incidence angle of the atomizer determine the flatness of the billets. With four atomizers positioned at eccentric distances of 141, 245, 316, and 375 mm; an incidence angle of 45°; scanning angles of 8°, 5°, 3°, and 1°; and a drawing speed of 0.1 mm/s, it is possible to produce large billets with a diameter of 800 mm. The pore distribution in the billet is characterized by a higher concentration at the periphery and a lower concentration at the center, with a tendency for pores to be more prevalent in the initial stage and decrease in the later stages.

## 1. Introduction

With the development of the aerospace industry, the demand for large, high-strength Al-Zn-Mg-Cu-Zr alloys has become increasingly urgent. However, alloys prepared using traditional semicontinuous casting methods can no longer meet these requirements [1,2,3]. Spray-forming technology combines the atomization process of molten metal with the deposition-forming process to directly produce billets, making it an advanced method for material preparation [4,5]. Compared to semi-continuous casting processes, this technology offers a higher cooling rate, which addresses issues such as low solid solubility, macrosegregation, and coarse structures [6,7,8]. It meets the production demands for large-sized, high-performance aluminum alloy components and is a crucial method for preparing Al-Zn-Mg-Cu-Zr alloys [9,10,11]. During the atomization process, the coverage of a single atomizer is relatively small, making it difficult to form large billets [12]. Therefore, the collaborative operation of multiple atomizers is necessary [13], and the coordination parameters between these atomizers have become a key focus in research on preparing large-sized billets.

The spray-forming process is complex and is influenced by numerous factors. As a result, dozens or even hundreds of process experiments are often required to determine the optimal process parameters and produce billets with regular shapes and specific dimensions [14]. To reduce costs and improve efficiency, many researchers have employed computer numerical simulation techniques to predict the jet deposition process [15]. Zhang et al. [16] conducted a numerical study on the deposition process in twin-atomizer spray forming. Their study revealed that the atomizer scan and substrate rotation frequencies are crucial factors affecting the quality of the molten droplets and the shape of the billets. When the common period exceeded 10, the surface quality distribution of the deposited billets was the most uniform. Cui et al. [17] simulated the process of preparing clad deposits under different nozzle spacing conditions and found that the smaller the distance between the nozzles, the thicker was the gradient zone between the two layers. Mi et al. [18] developed a model to simulate the porosity distribution inside a spray-formed ring preform. The model indicated that the porosity at the edges of the billet was significantly higher than that at the central part. Pryds et al. [19] developed a deposition billet growth model and calculated the growth process of the model under different gas–liquid ratios. The results showed that the height of the preform decreases with increasing gas-to-metal ratio, for example, at the centerline, and the surface temperature was found to decrease linearly with increasing gas-to-metal ratio regardless of the position.

At present, most simulations and experiments only consider scenarios involving one or two atomizers [20]. Such configurations are incapable of producing large billets exceeding 800 mm, making it difficult to meet the growing industrial demands. Therefore, simulations to model the multiple atomizer spray forming process and analyze the influence of the atomizer distribution on the shape of the billet can provide theoretical support for the preparation of large-scale billets [21]. This study aims to design a configuration method for atomizers that enables the fabrication of large billets with diameters exceeding 800 mm.

## 2. Methods

### 2.1. Shape Model

After the alloy melt flows out, it is atomized into small droplets by gas ejected from the nozzle. Under normal circumstances, atomized droplets are uniformly distributed within the spray cone and the radial mass distribution exhibits a functional relationship resembling an approximate Gaussian distribution [22,23,24,25]. The creation of the shape model for the deposited billet was also based on this functional relationship, as shown in Equation (1) [26]:

(1) $$D\left(R\right)=M\cdot \exp\left(HR^{2}\right)$$

In this context, *M* represents the mass density at the center of the spray cone, which is numerically equal to the peak value of the Gaussian distribution. The variable *H* represents the height distribution coefficient of the jet density. *R* denotes the distance from any point on the deposition surface to the central axis of the spray cone.

Equation (1) was used to calculate the radial distribution of the droplet spray density at a specified distance. However, in the actual spraying process, the distance traveled by the droplets to the surface of the billet is uncertain. In the initial stage, the spray distance changed constantly. Therefore, using the above formula directly in the calculations can lead to certain deviations. The optimized formula based on this is

(2) $$m\left(R,h\right)=a_{0}\cdot {\left(\frac{h_{0}}{h}\right)}^{2}\cdot \exp\left[-b_{0}\cdot {\left(\frac{h_{0}}{h}\right)}^{2}\cdot R^{2}\right]$$

The actual deposition rate *D*(*R*) is:

(3) $$D\left(r,h\right)=\eta \cdot m\left(r,h\right)$$

where *η* represents the adhesion efficiency within the spray cone. In accordance with the findings reported by Z. Djurić [27], the value of *η* is generally taken to lie within the range of 0.94–0.96. Furthermore, the deposition yield determined experimentally in the present study is 91%, which indicates that, with the exception of the solid alloy particles entrained by the gas stream, the majority of the droplets are able to deposit onto the substrate. Accordingly, a value of *η* = 0.96 is adopted in the present work.

Based on the actual operating conditions, the following assumptions were made for the billet shape prediction model [28]: (1) the droplet density distribution within the spray cone conforms to the distribution Equation (1); (2) other airflows do not influence the accelerated motion of the droplets, meaning that their flight state remains unchanged and the droplets consistently travel in a straight line; and (3) the rotational speed of the substrate is significantly greater than the growth rate of the deposition surface.

**Figure 1:**
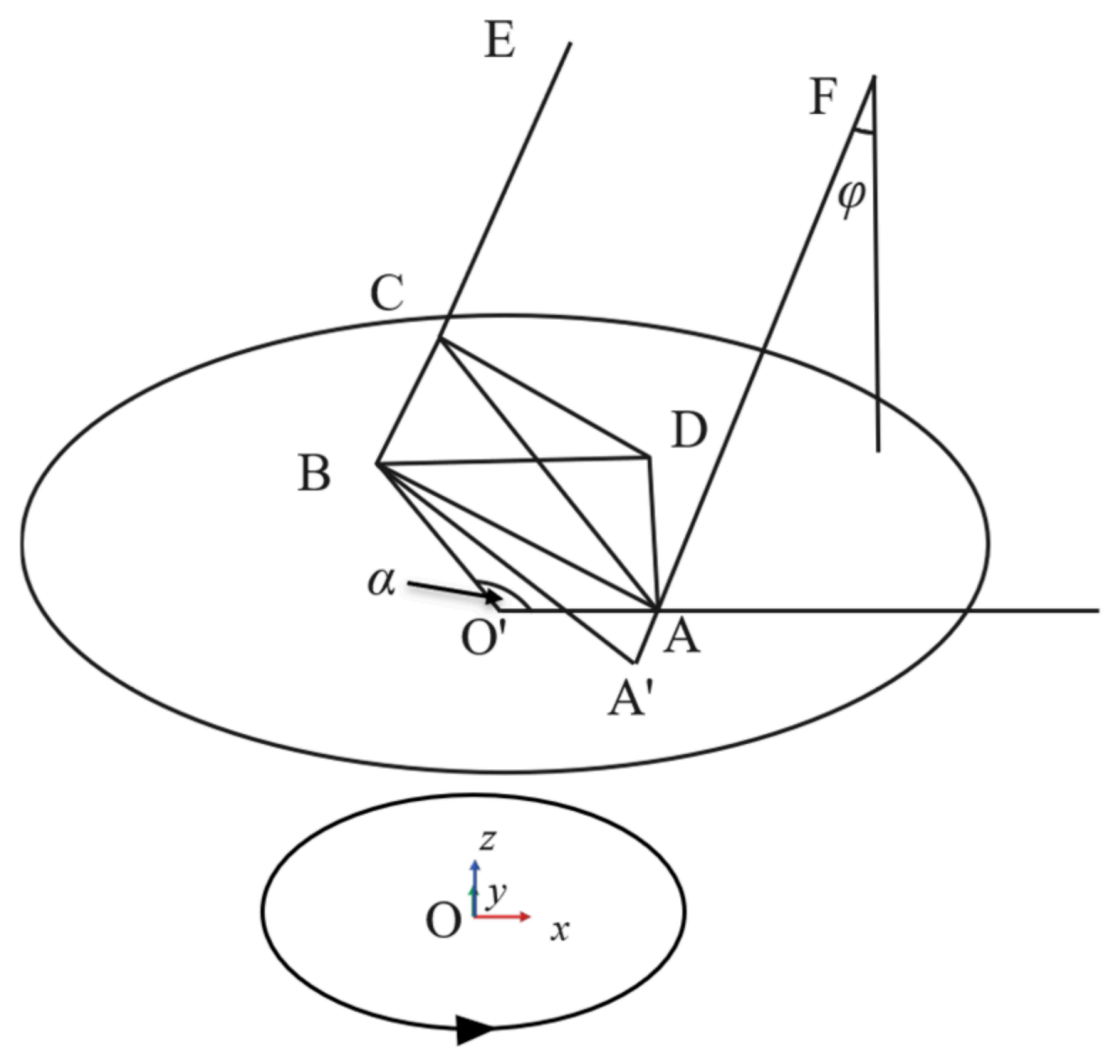
Schematic diagram of the distance relationship between an arbitrary point *B* on the surface of the billet and the axis of the spray cone.

Under actual operating conditions, the substrate rotates and moves downward, while the gas flow is injected toward the substrate surface at a certain incidence angle (0–90°), making a cylindrical coordinate system appropriate. The base plane of the cylindrical coordinate system is defined on the substrate surface, and the *z*-axis coincides with the substrate axis. Figure 1 illustrates the spatial geometric relationships between various points on the surface of the billet. The vertical distance from any point *B*(*z*,*r*,*α*) on the substrate surface to the centerline of the spray cone can be represented by *BA*′ in Figure 1. The green circle in the figure denotes the horizontal plane where point *B*(*z*,*r*,*α*) is located, with point O′ being the axisymmetric center of this circle. Point *A* is the intersection of the spray cone centerline with the horizontal plane where point *B* is located. Point *F* represents the position of the liquid guide tube outlet, and *FA* is the line where the spray cone center is located. Symbol *ϕ* denotes the incidence angle of the atomizer. In the horizontal plane where point *B* is located, a line *DA* is drawn through point *A* such that *DA* is perpendicular to *AO*, and a line *BD* is drawn such that *BD* is perpendicular to *AD*. To construct a spatial line *BE* parallel to *FA* through point *B*, a perpendicular line was drawn from point *D* to line *BE*, intersecting *BE* at point *C*. Connect *AC*, and then draw *BA*′ parallel to *CA* through point *B*, intersecting line *AF* at point *A*′. According to the geometric positional relationship, *AC* is perpendicular to *AF*, so *BA*′ is also perpendicular to *AF*·*BA*′(=*AC*) should represent the distance from point *B* to the center of the spray cone, which corresponds to the value *R* in Equation (2), while *A*′*F* corresponds to *h* in the equation. If the initial eccentric distance at the onset of atomization is denoted as *l*_0_, then *AO*′ can be calculated using Equation (4).

(4) $$AO^{\prime }=l_{0}-\left(h_{1}-h_{2}\right)\cdot \mathrm{ctg}\left(\phi \right)$$

where *h*_1_ represents the downward movement distance of the substrate, *h*_1_ = *vt*, *v* is the downward movement speed of the substrate, and *h*_2_ is the growth height on the substrate surface, expressed as *h*_2_ = *z*(*B*).

The distance *R_B_* from point *B* to the spray center at any given moment can be deduced as

(5) $$R_{B}=\sqrt{r^{2}\sin^{2}\left(\alpha +\theta \right)+{\left[l_{0}-\left(\mathrm{vt}-z\right)\mathrm{tg}\phi -r\cos\left(\alpha +\theta \right)\right]}^{2}\cos^{2}\phi }$$

where *z* is the axial coordinate of point *B*, *r* is the radial coordinate of point *B*, and *θ* is the angle through which the substrate rotates. If the initial spray distance is set as *h*_0_, then the actual spray distance *h* at any given time can be expressed as:

(6) $$h=\mathrm{FA}+AA^{\prime }=\frac{h_{0}\cos\phi +\mathrm{vt}-z}{\cos\phi }+\left[l_{0}-\left(\mathrm{vt}-z\right)\mathrm{tg}\phi -r\cos\left(\alpha +\theta \right)\right]\sin\phi$$

The values of *R_B_* and *h* calculated from the above equation are substituted into *R* and *h* of Equation (2). The resulting spray rate *m*(*R*, *h*) was then substituted into Equation (3) to obtain the deposition rate at point *B* at any given time. Because the substrate rotated at a high speed during the deposition process, the actual distance from point *B* to the symmetric center of the spray cone continuously changed over time. Therefore, the deposition rate at any point *B* during the deposition process is a function of time, and the direction of the spray cone deposited at point *B* continuously changes.

**Figure 2:**
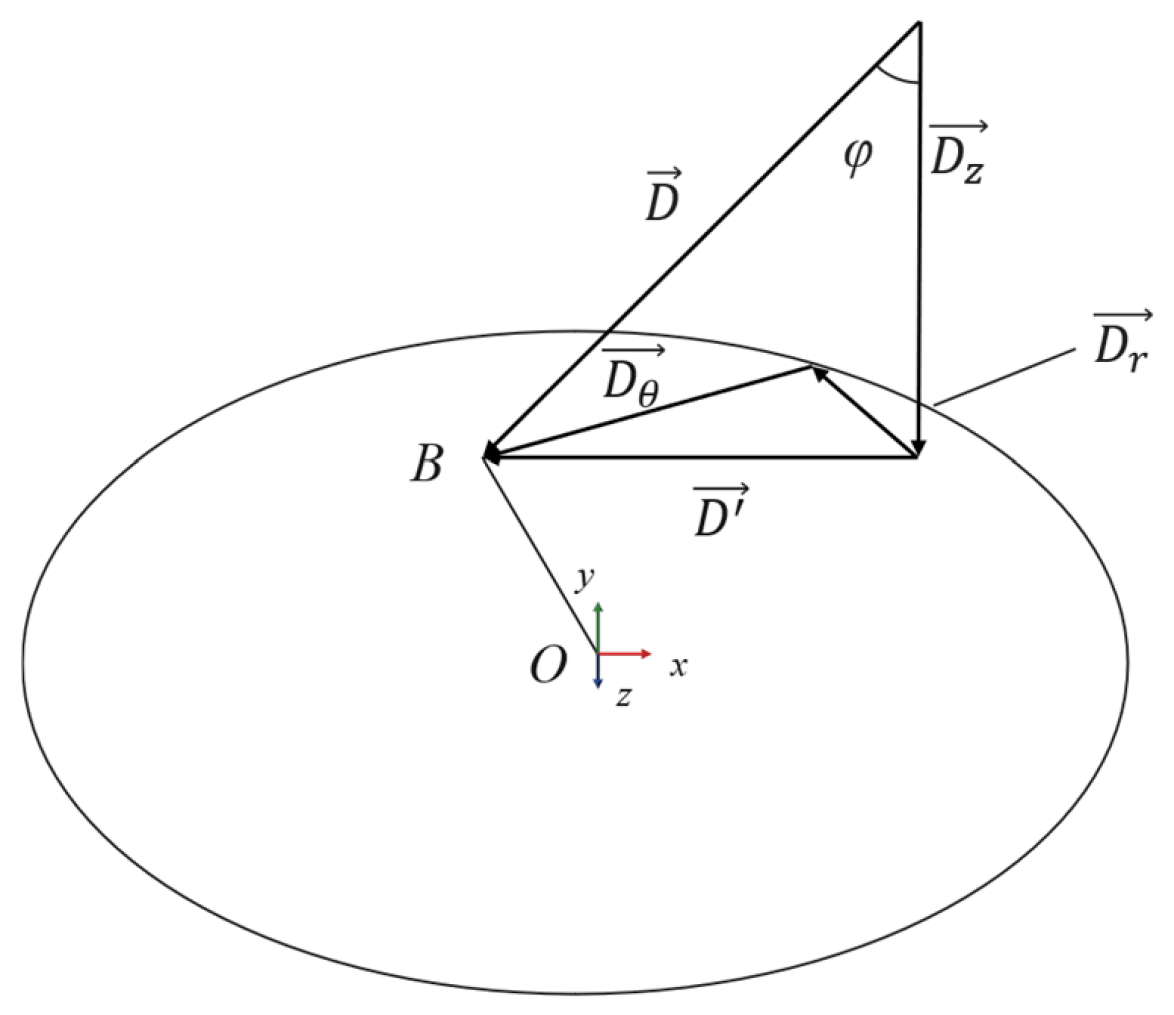
Vector orientation diagram of the spray.

For computational convenience, this study decomposed the spray cone into two components: one perpendicular to the surface of the substrate and the other parallel to the surface of the substrate, denoted as $\vec{D_{Z}}$ and $\vec{D^{\prime }}$, respectively. Subsequently, the component parallel to the surface, $\vec{D^{\prime }}$, is further divided into radial component $\vec{D_{r}}$ and *θ* component perpendicular to the radial direction, as illustrated in Figure 2.

(7) $$\left\{\begin{array}{c} \left|\vec{D_{Z}}\right|=\left|\vec{D}\right|\times \cos\phi \\ \left|\vec{D_{r}}\right|=\left|\vec{D}\right|\times \sin\phi \times \cos\left(\alpha +\theta \right)\\ \left|\vec{D_{\theta }}\right|=\left|\vec{D}\right|\times \sin\phi \times \sin\left(\alpha +\theta \right) \end{array}\right.$$

The change in coordinates for any point *B* on the substrate is:

(8) $$\left\{\begin{array}{c} z\left(B\right)=\int \left|\vec{D_{Z}}\right|\cdot \mathrm{dt}\\ r\left(B\right)=r_{0}\left(B\right)+\int \left|\vec{D_{r}}\right|\cdot \mathrm{dt}\\ \alpha \left(B\right)=\alpha_{0}\left(B\right)+\int \left|\vec{D_{\theta }}\right|\cdot \mathrm{dt} \end{array}\right.$$

During the deposition process, the substrate rotated continuously. At any point *B*, when the angular coordinate is *α* + *θ*, the increment in *θ* caused by this is exactly equal in magnitude, but opposite in direction to the increment in *θ* caused by point *B* at the 2*π*-*α*-*θ* position, thereby canceling each other out. Therefore, in the calculations, changes in the *θ* direction can be disregarded. Using Equation (8), the coordinate changes in all points on the surface of the billet can be calculated, yielding the contour shape of the billet at any given moment. The substrate rotates at *ω* = 80 rpm, i.e., one revolution takes *T* = 0.75 s, while the deposition surface grows at the withdrawal rate of the substrate (0.1–1 mm/s). The thickness deposited in a single revolution is therefore only *Δz* = *vT* = 0.075–0.75 mm, which is 10^−4^–10^−3^ of the billet diameter (200–800 mm) and is smaller than both the semi-solid (mushy) layer thickness on the deposition surface and the thermal diffusion length within one revolution, √(*aT*) ≈ 5.5 mm. The tangential velocity at the billet periphery, *u* = *ωR* ≈ 0.84–3.35 m/s, exceeds the surface growth velocity by three to four orders of magnitude (*u*/*v* ≈ 8 × 10^2^–3 × 10^4^). Consequently, every point at a given radius experiences essentially the same sequence of spray conditions per revolution, and the azimuthal non-uniformities introduced within one revolution are re-melted and levelled by the liquid film before the next revolution. The deposit is therefore a solid of revolution, in agreement with the criterion that a uniform mass distribution is obtained when the substrate rotation is fast relative to the periodic deposition pattern [16], and with previous axisymmetric billet-shape models on rotating substrates [18,28,29]. Depositing a billet of height *H* requires *N* = *H*/*Δz* ≈ 400–4000 revolutions, so that the final shape is the accumulation of a very large number of thin, fully mixed layers. Accordingly, the 3-D deposition problem is reduced to a 2-D axisymmetric (*r*–*z*) formulation.

This study employs the “Birth & Death” function in ANSYS 2021 R1 (ANSYS Inc., Canonsburg, PA, USA) to simulate the deposition process. This function enables the simulation of changes in the deposited billet during atomization and facilitates the analysis of various special operating conditions encountered in industrial production, making it an effective approach for investigating this issue. In practical applications, it is first necessary to complete the modeling of all the elements in the PREP7 preprocessor, including those to be controlled. This requirement is primarily determined by the characteristics of the ANSYS system, which do not allow elements to be directly added or removed during the SOLU solution phase. Instead, model modification must be carried out using the two control commands, EALIVE and EKILL. Additionally, it should be noted that during this stage, the units that are eliminated are not removed. Instead, the software employs a reduction factor to infinitely shrink them, resulting in all their attribute values being zero. Similarly, the activation process involves nullifying the effect of the reduction factor to restore units to their normal state.

Based on the above points, it is necessary to include all elements of the billet in the pre-processing PERP7 module to calculate the birth and death elements in the deposition process. At the initial stage of the deposition process calculation, these elements were treated as “killed.” Only elements that meet specific condition commands can be activated and incorporated into the calculation and solving process, ultimately allowing for the simulation and calculation of the entire deposition process. In summary, the “birth and death element method” can be used in ANSYS software to implement the spray forming process. A thermal equilibrium approach can also be employed to discretely solve the process under finite element conditions, thereby achieving temperature analysis.

### 2.2. Atomisation Model

**Figure 3:**
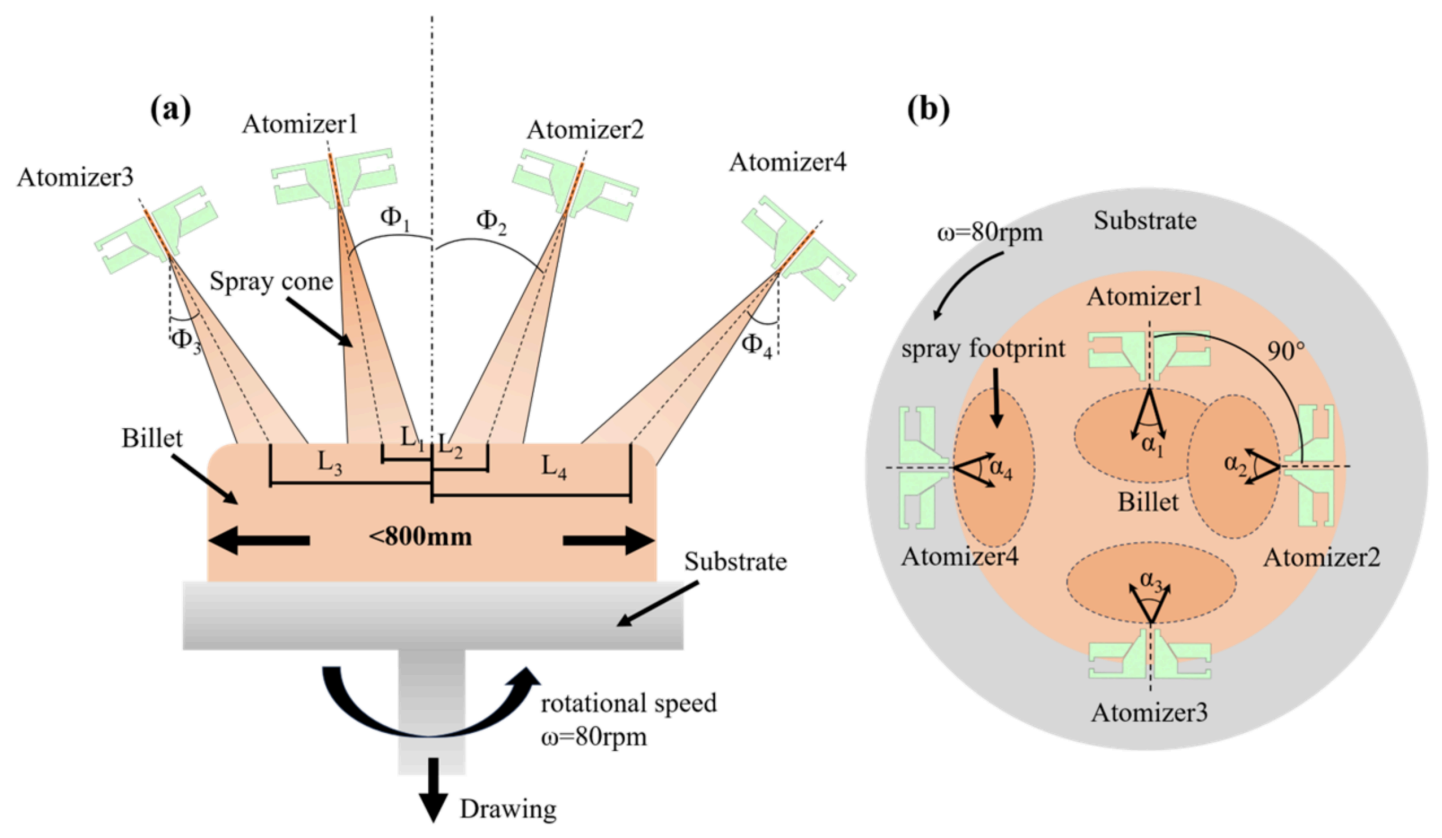
Schematic diagram of the atomizer arrangement: (**a**) side view; (**b**) top view.

A close-coupled gas atomizer (Jiangsu Haoran Spray Forming Co., Ltd., Zhenjiang, China) was employed in this study, and the nozzle included an angle of 60°. The outlet length of the liquid delivery tube was 2 mm and the inner diameter was 3 mm. Four atomizers were positioned in four directions with a 90° angle between them. The scanning angle is defined as the angle through which the atomizer axis is rotated to and fro in the horizontal plane relative to a reference orientation, thereby enabling the spray cone to scan circumferentially along the billet surface. A schematic representation of this arrangement is shown in Figure 3.

This study primarily investigates the effects of the eccentric distance, incidence angle, scanning angle of the four atomizers, and substrate drawing speed on the morphology of the billet. The effects of the individual factors on the billet shape are as follows. Eccentric distance: For a given drawing speed, an optimum eccentric distance exists; any deviation from it leads to a non-uniform radial distribution of the metal liquid, which in turn produces pits on the top surface, annular peaks, and shielding of the side surface. The lower the drawing speed, the larger the optimum eccentric distance and the corresponding difference between the eccentric distances. Incidence angle: Increasing the incidence angle markedly improves the flatness of the billet but raises its porosity; it should therefore not exceed 20°. Scanning angle: Moderately increasing the swing angle helps to level the top surface, but exceeding a certain limit causes an excessive flux at the center of the spray region, which in turn leads to undulations at the billet center. Drawing speed: The drawing speed determines the maximum billet diameter attainable, and the slower the speed, the larger the diameter. However, the larger the billet, the narrower the process window and the more difficult it is to control. No mold is employed in this process, and the billet shape is determined by the atomizer arrangement and the drawing speed.

Four drawing speeds were set: 1, 0.5, 0.25, and 0.1 mm/s. Various atomizer arrangement combinations were configured for each drawing speed, as shown in Table 1. All arrangements are numbered in order to facilitate the later expression, take “3-2: L^0.25^_21_” as an example, “3-2” represents the third arrangement at the third drawing speed (0.25 mm/s); “L” represents eccentric distance; “0.25” represents drawing speed; “2” represents the second arrangement at 0.25 drawing speed; “1” represents atomizer No. 1.

## 3. Results and Discussions

### 3.1. Billet Shape

**Figure 4:**
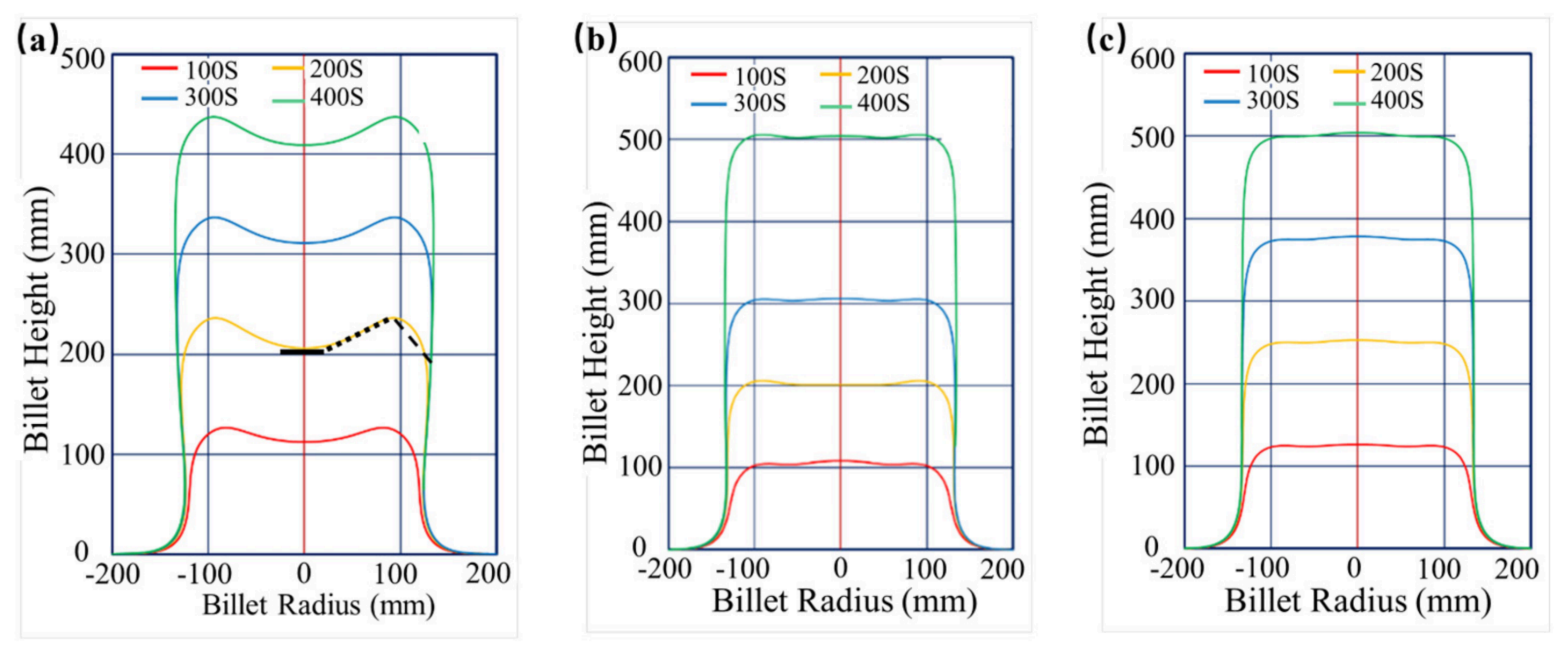
Morphology of the billet at different eccentric distance under a drawing rate of 1 mm/s: (**a**) arrangement 1−1; (**b**) arrangement 1−2; (**c**) arrangement 1−3. The solid line in (**a**) marks the landing position of the metal liquid ejected from the inner atomizers, and the dashed line marks the landing position of the metal liquid ejected from the outer atomizers. They show where the droplets from each atomizer are deposited on the top surface of the billet.

The shape of the billet when the drawing speed was 1 mm/s and the eccentric distances were 50, 71, 87, and 100 is shown in Figure 4. During the initial stage of deposition, small pits began to appear as early as 100 s. As the deposition process continued, these pits gradually enlarged, while the diameter of the upper part of the billet increased slightly. At 200 s, the pits reached their maximum size, after which they slightly leveled during the deposition process, although the diameter of the upper billet continued to increase marginally. Concurrent with the formation of pits, an annular ridge developed around the periphery of the billet. The curvature at the ridge crest reached its maximum value at 200 s and remained nearly unchanged thereafter.

When pits and ring peaks appear in the middle of the billet, there are two effects on the deposition process. For the inner atomizer, most of the metal liquid is deposited in the fine dashed area in Figure 4a, while a smaller portion is deposited in the thick solid line area. At this point, the actual incidence angle of most of the metal liquid increases, making it difficult for some of the liquid to adhere to the surface of the billet, leading to an increase in the porosity of the billet. Because only a small portion of the metal liquid is incident at the center of the pit, the pit expands further. When the pit expands to a certain extent and there is sufficient metal liquid sprayed on the fine dashed line without falling off, it will then drop to the thick solid line area, causing the growth of the pit to stop and maintain this steady state. For the external atomizer, a small portion of the metal liquid was sprayed onto the thin dotted area, whereas the majority was sprayed onto the thick dotted area. The metal liquid sprayed onto the thin dotted area, owing to the excessive incident angle, largely falls directly onto the thick solid area, inhibiting the growth of the pit. Conversely, the metal liquid sprayed onto the thick dotted area mostly adhered to the surface of the billet, with a small portion sliding outward and adhering to the lower sidewalls, resulting in an increase in the diameter of the upper billet. The appearance of the annular peak indicates the excessive deposition of the metal liquid in that area. Upon analysis, the likely cause was the small difference in the eccentric distance of the atomizer. By increasing the eccentric distance of the outer atomizer, a noticeable improvement in the shape of the billet was observed. During the deposition of the billet, the diameter remained stable, and the top surface experienced slight disturbances, initially showing small protrusions, followed by the emergence of small annular pits. Based on this, the uniformity of the metal liquid was improved by increasing the eccentric distance of the outer atomizer and the scanning angle of the inner atomizer, leading to an enhanced flatness of the billet, as shown in Figure 4b. An attempt was made to increase the coverage area of the molten metal stream by increasing the eccentric distance of the atomizers to obtain an ingot with a larger diameter, as shown in Figure 4c. However, the effect was not statistically significant. At this stage, the growth rate of the billet could no longer keep pace with the downward movement of the substrate, resulting in a noticeable decrease in the height of the billet.

**Figure 5:**
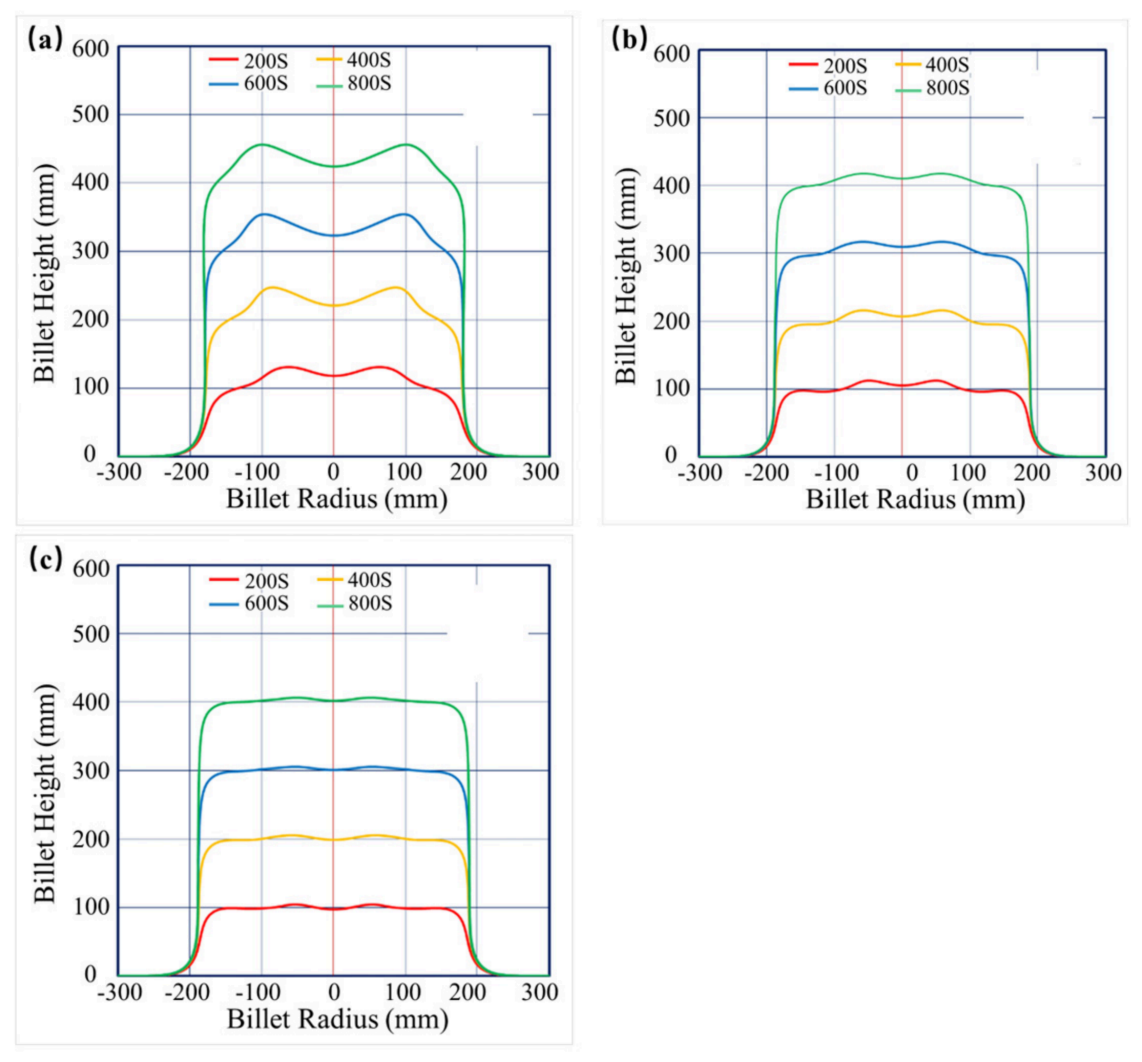
Morphology of the billet at different eccentric distance under a drawing rate of 0.5 mm/s: (**a**) arrangement 2−1; (**b**) arrangement 2−2; (**c**) arrangement 2−3.

It was difficult to obtain billets with larger diameters at a drawing speed of 1 mm/s. However, if the drawing speed of the substrate is reduced, billets with larger diameters can be produced by increasing the spacing between atomizers and the maximum eccentric distance. As shown in Figure 5a, after reducing the drawing speed and increasing the eccentricity distance of the atomizer, the diameter of the billet increased significantly. However, maintaining the surface flatness of the billet is more challenging. Under these process parameters, the top-surface flatness of the billet was relatively low, whereas the flatness of the side surfaces was comparatively high.

Its diameter was approximately 180–200 mm. The billet can be approximately divided into four regions according to the radial distance: the region from 0 to 50 mm is associated with atomizer no. 1, 50–100 mm with atomizer no. 2, 100–150 mm with atomizer no. 3, and the region beyond 150 mm with atomizer no. 4. By modifying the process parameters, atomizer no. 2 was moved outward, increasing its eccentric distance to 120. Atomizer No. 3 was also shifted outward to prevent the excessive accumulation of molten metal in the region with a diameter of 100–120 mm. Meanwhile, atomizer No. 1 was moved outward to avoid insufficient molten metal in the 0–70 mm diameter region. Consequently, the billet shape shown in Figure 5b was obtained. The flatness of the billet top surface significantly improved; however, a small annular peak still appeared at a diameter of approximately 50 mm. Further increasing the eccentric distance of Atomizer No. 1 reduced the ring-shaped peak at the 50 mm diameter position, and the flatness of the billet top surface improved, as shown in Figure 5c; however, a small depression appeared at the center. At this stage, a relatively good simulation result has already been obtained at a pulling rate of 0.5 mm/s, indicating that further improvement of the billet morphology may no longer be achievable by adjusting the atomizer eccentric distance alone.

**Figure 6:**
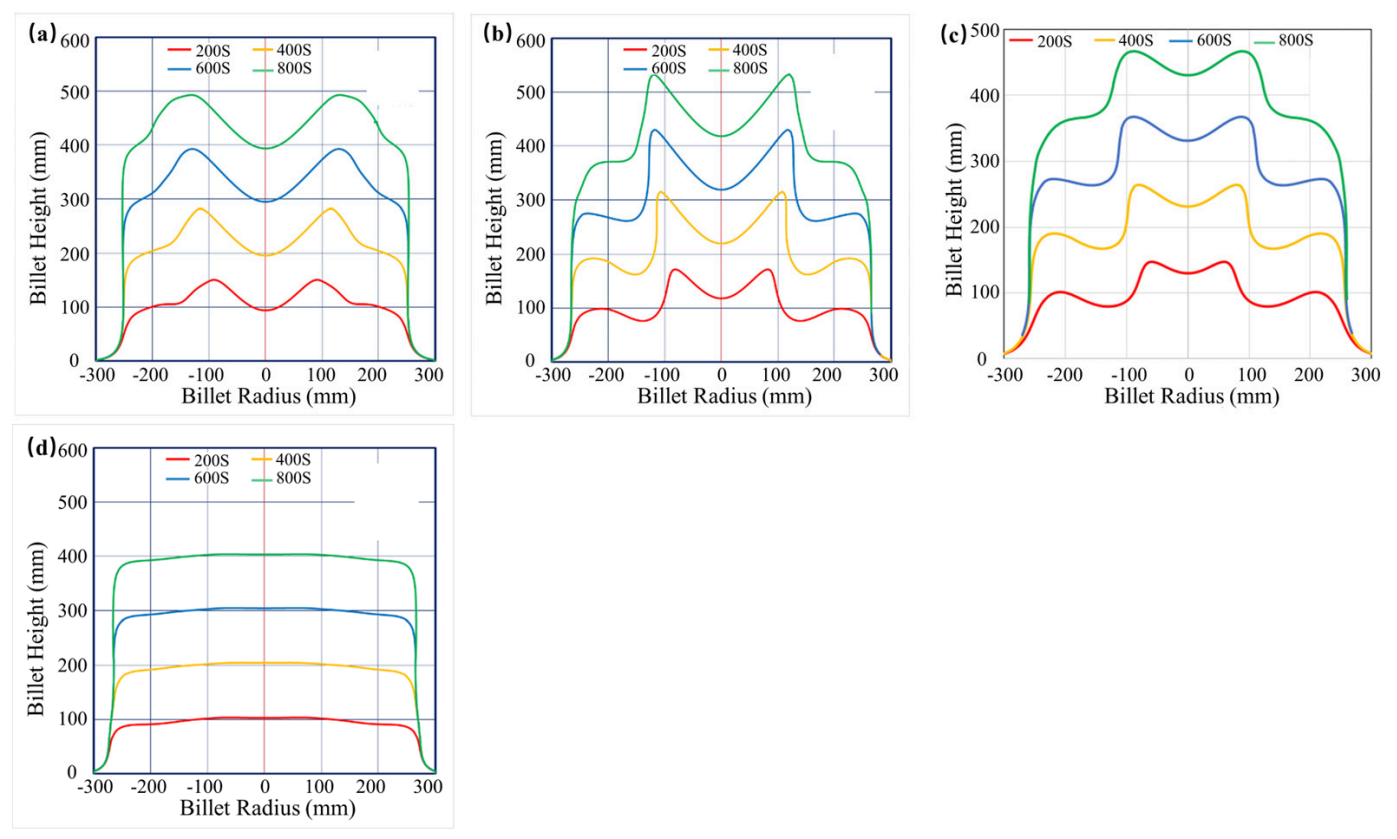
Morphology of the billet at different eccentric distance under a drawing rate of 0.25 mm/s: (**a**) arrangement 3−1; (**b**) arrangement 3−2; (**c**) arrangement 3−3; (**d**) arrangement 3−4.

By further reducing the drawing speed of the substrate to 0.25 mm/s, a billet with a larger diameter was obtained. However, the top of the billet exhibited pronounced undulations, as shown in Figure 6a. The resulting billet shape obtained by decreasing the eccentric distance of Atomizer No. 1 and increasing the eccentricities of Atomizers No. 2 and No. 3 is shown in Figure 6b. It can be observed that merely adjusting the eccentric distance is insufficient for regularizing the billet morphology. An attempt was made to improve billet surface flatness by increasing the scanning angle. The scanning angles of the atomizers were adjusted to 7-5-5-5°, producing the billet shown in Figure 6c. Modification of the scanning angles also failed to eliminate depressions and protrusions on the top surface of the billet. In summary, the only viable option is to increase the angle of incidence. However, according to empirical experience, an increase in the incidence angle tends to increase the porosity of the billet. When the incidence angle of the atomizer was adjusted to 45°, a well-formed billet with a diameter exceeding 500 mm was obtained, as shown in Figure 6d. If the subsequent densification processing is applied to the spray-formed billet, dense alloy billets can be produced.

**Figure 7:**
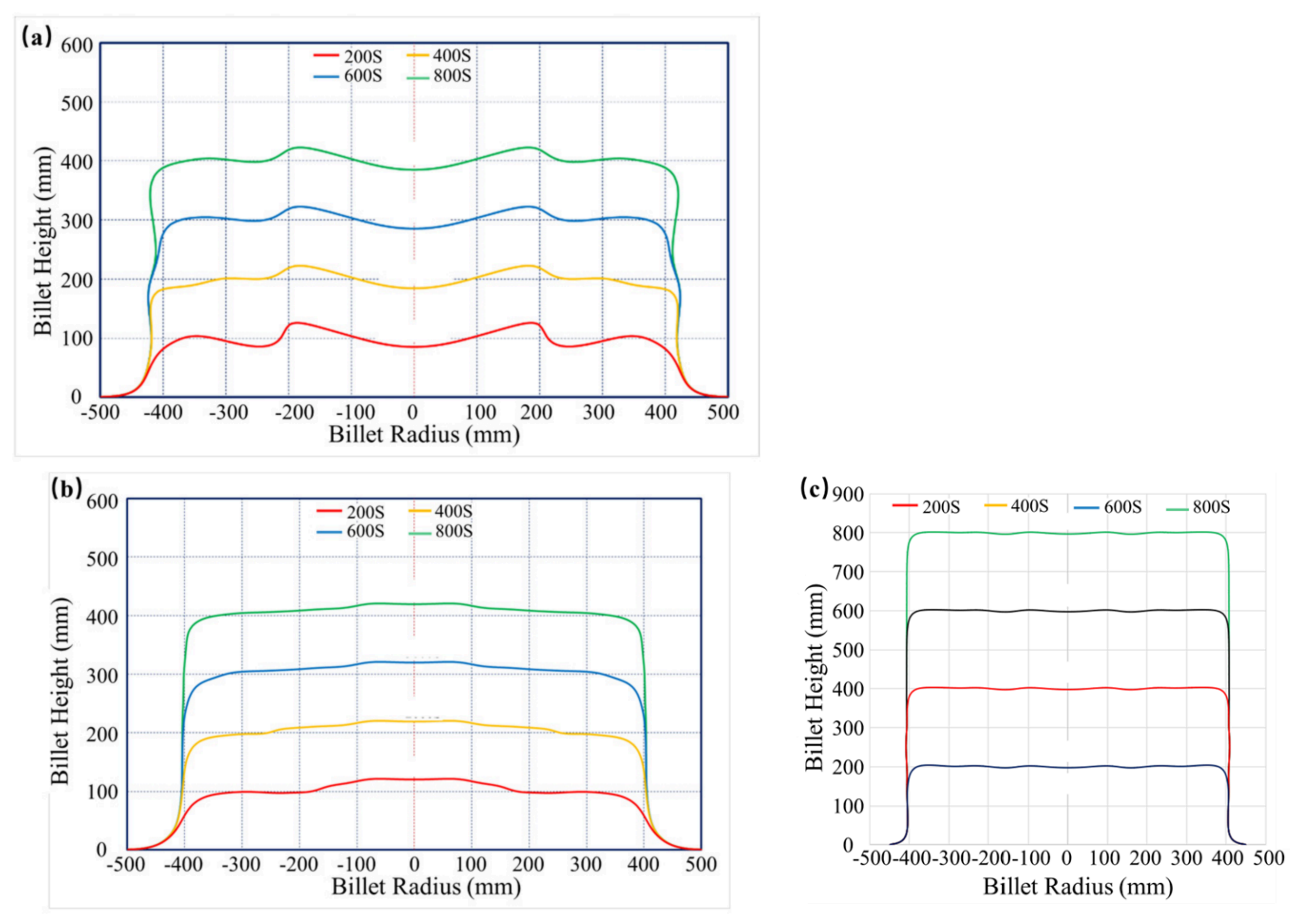
Morphology of the billet at different eccentric distance under a drawing rate of 0.1 mm/s: (**a**) arrangement 4−1; (**b**) arrangement 4−2; (**c**) arrangement 4−3.

The drawing speed of the substrate was further reduced to 0.1 mm/s, and the eccentric distances of the atomizers, together with the differences between the eccentric distances of adjacent atomizers, were increased, yielding the billet shown in Figure 7a. At this stage, the billet diameter reaches 800 mm. A depression appeared at the center of the billet, while a protrusion was observed 200 mm from the center, and the side surface of the billet exhibited significant undulation. To optimize the morphology of the billet top, the eccentric distance and scanning angle of Atomizer No. 1 were increased, whereas the eccentric distance of Atomizer No. 2 was reduced and its scanning angle increased. This adjustment allowed for more uniform metal flow in the central region. Simultaneously, the eccentric distances of atomizers No. 3 and No. 4 were reduced to fill the localized depressions on the top surface of the billet. The resulting billet morphology is shown in Figure 7b. It can be observed that although the diameter of the billet has decreased, its flatness has significantly improved. Further precise optimization and adjustment of the atomizer parameters ultimately resulted in a standard cylindrical billet, as shown in Figure 7c.

**Figure 8:**
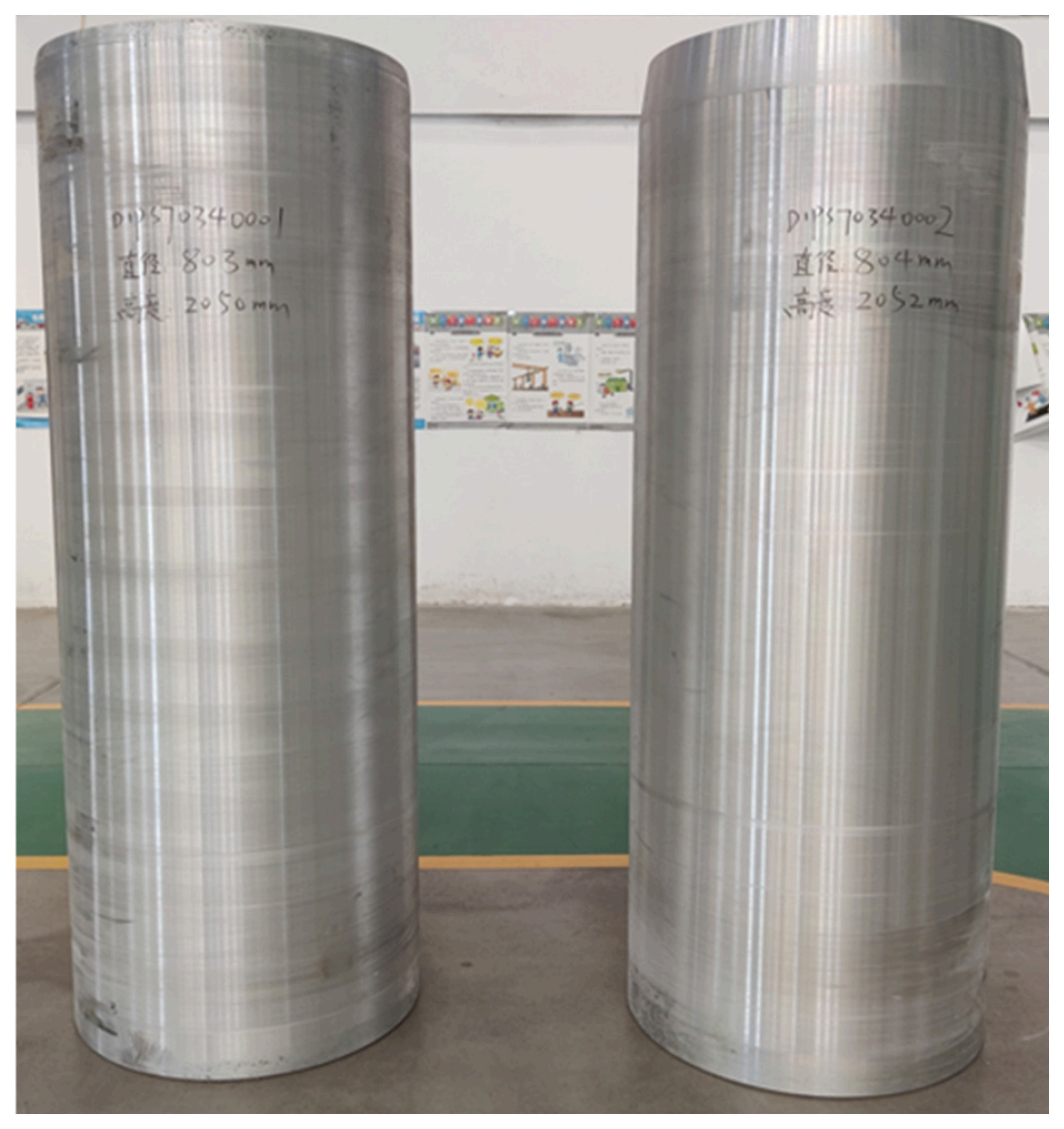
Morphology of the billet under the 4−3 arrangement. The Chinese annotations on the two billets record the measured billet dimensions; the billet in the left panel is 803 mm in diameter and the billet in the right panel is 804 mm.

The optimized configuration in Figure 7c was verified by spray experiments, and the results are shown in Figure 8. The alloy composition used was Al-11.3Zn-2.45Mg-1.5Cu-0.12Zr-0.05Fe (wt%), supplied by Jiangsu Haoran Spray Forming Co., Ltd. (Zhenjiang, China), with the temperature in the liquid delivery tube being approximately 780 °C. The substrate was rotated at 80 rpm, the melt flow rate was 50 kg/min, and the atomizing gas pressure and temperature were 1 MPa and 21 °C, respectively. Four close-coupled gas atomizers were employed, with an arrangement of eccentric distances of 141.4-245-316.2-374.2 mm, an incidence angle of 45° and a scanning angle of 8-5-3-1°. The shape of the resulting billet is shown in Figure 8. The billet diameters are 803 mm (left) and 804 mm (right), and the flatness of the top surface is 1 mm. The flatness is calculated as follows: After excluding the edge region, 100 points were taken at equal intervals along the radial direction of the top surface. The height of each point was measured, and the arithmetic mean was determined. The mean was subtracted from each measured height, and the squared differences were averaged and then square-rooted; the resulting standard deviation is defined as the flatness of the top surface. By comparing the predicted billet shape in Figure 7c (diameter 817 mm, flatness 1.8 mm) with that obtained experimentally, the relative error in diameter is found to be about 1.6%, and the flatness of both billets is satisfactory. These results indicate that the atomizer process parameters optimized by numerical simulation are applicable to guiding the actual billet fabrication.

### 3.2. Pores in the Billet

**Figure 9:**
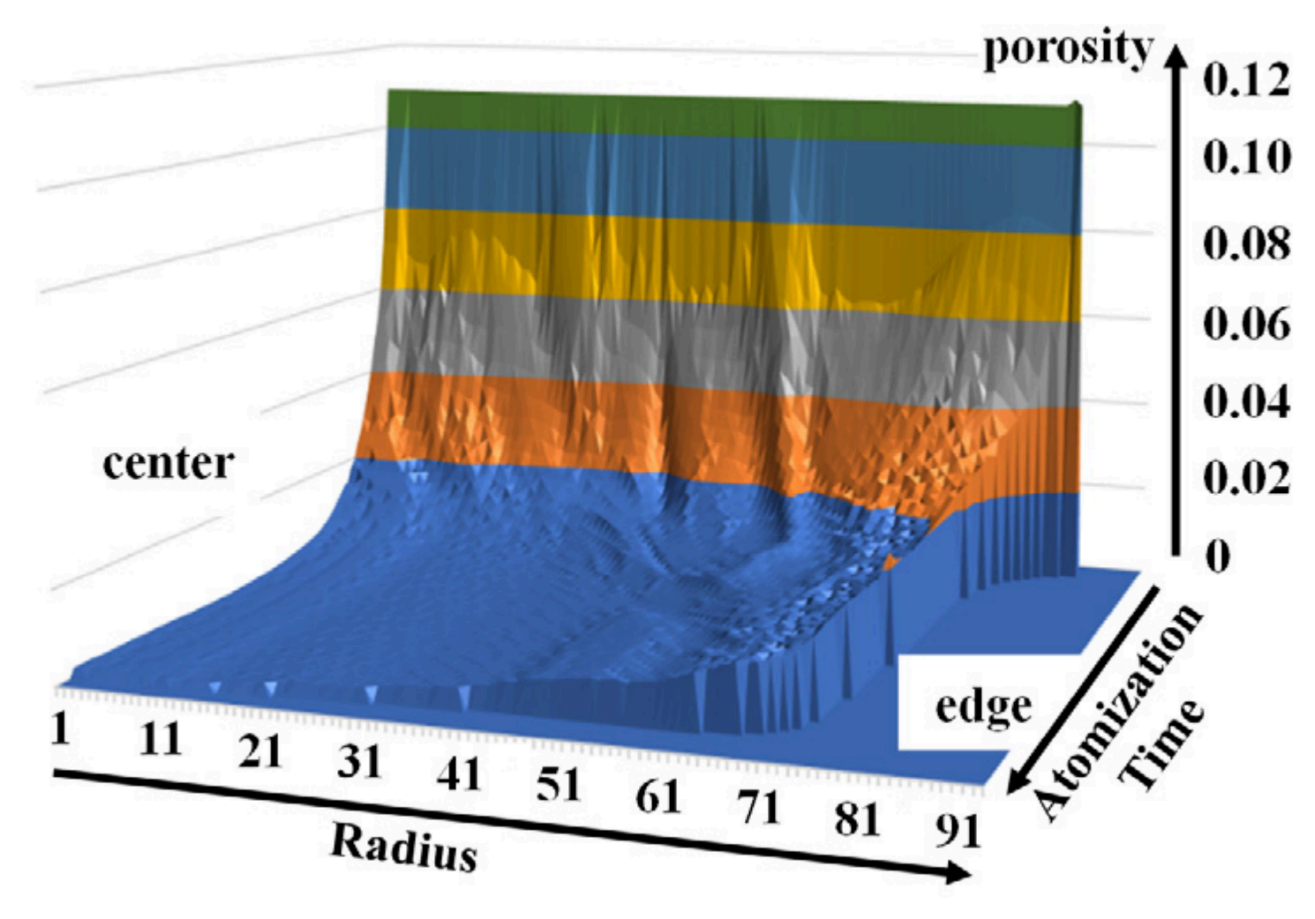
The porosity of billet under the 3−4 arrangement. The different colours are used to distinguish the different porosity ranges.

Materials produced by spray forming are classified as porous [30]. Because the feedstock during the deposition process primarily consists of semi-solid droplets, a relatively high porosity is generated and oxide films are readily formed [31]. Consequently, the billet often fails to achieve an ideal metallurgical structure and tends to exhibit a loose microstructure [32]. Therefore, the distribution of pores in the billet needs to be analyzed so as to guide the subsequent hot working process. Porosity is a commonly used index for evaluating the amount of pores in a microstructure, and it is defined as the volume (area) fraction (%) of voids within a unit volume (area). Simulating the porosity of the billet, analyzing the results, and predicting potential defects can provide valuable guidance for subsequent billet densification processes. During the deposition process, the billet rotates at a certain rotational speed, and droplets are deposited onto its surface at a specific inclination angle. Because the rotational speed of the billet is much higher than the growth rate of the deposition surface, half of the cross section passing through the central axis of the billet can be selected to represent the overall porosity distribution of the billet. Figure 9 shows the porosity distribution of the billet under the 3–4 arrangement conditions. The porosity of the billet exhibits the following two characteristics: (1) a higher porosity occurs at the interface between the billet and substrate, and (2) the porosity near the outer region of the billet is higher than that at the billet center.

**Figure 10:**
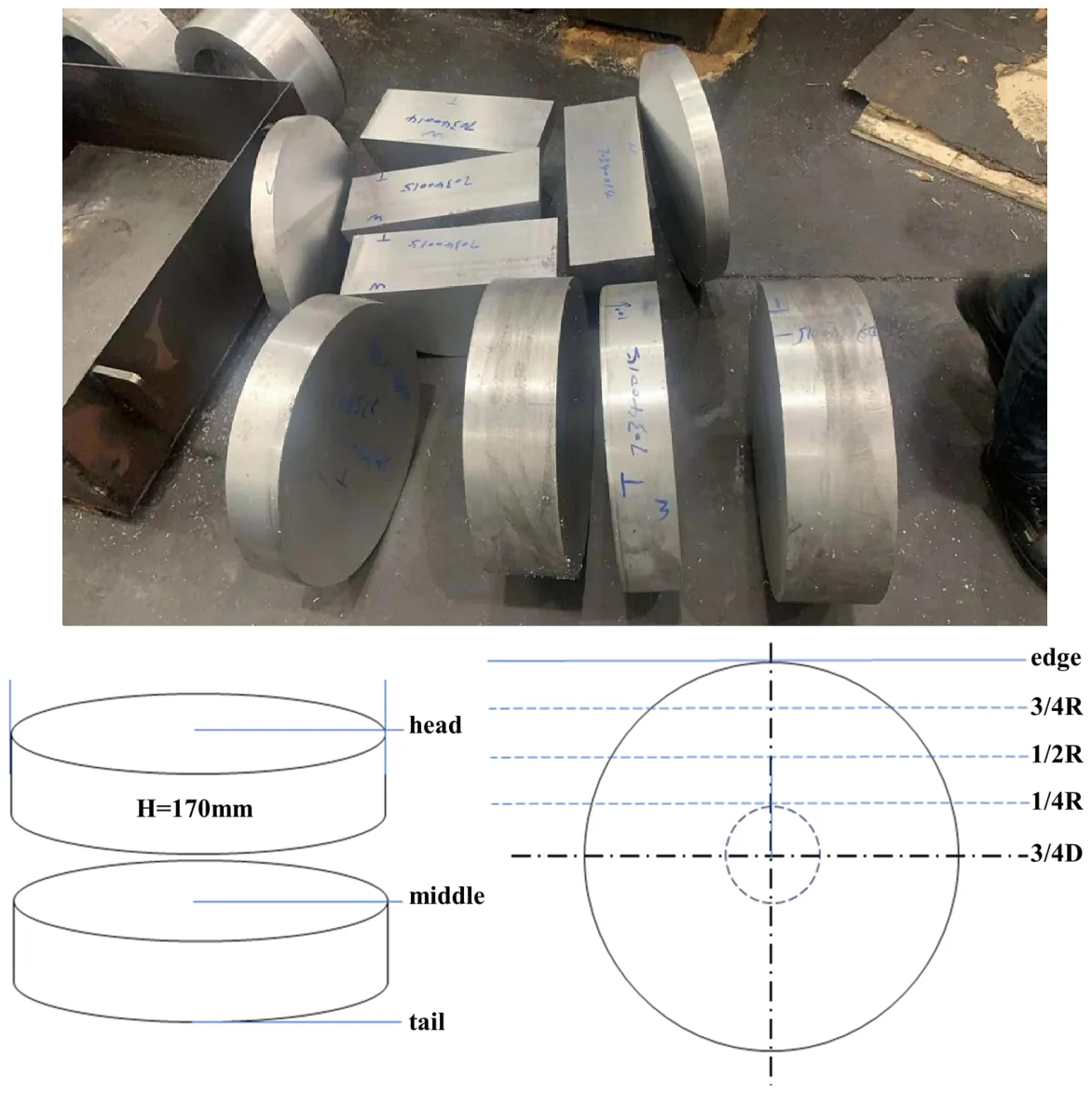
The sampling part of the billet. The different colours are used only to distinguish the individual parts of the sampling scheme, the dash-dot lines are the centre lines of the cross-sections, and the dashed lines mark the radial sampling positions (1/4R, 1/2R and 3/4R) and the billet edge.

As shown in Figure 10, the billet was sectioned, and samples were taken from the cross-sections of the head, middle, and tail regions. At each cross-section, specimens were extracted at radial positions of 1/4R, 1/2R, and 3/4R from the billet center, as well as from the central and edge regions. The pore distribution was analyzed through metallographic observations of the microstructure on a scanning electron microscope (SU5000, Hitachi High-Tech Corporation, Tokyo, Japan). The pore area fractions and pore sizes were measured on the micrographs with ImageJ 1.54r (National Institutes of Health, Bethesda, MD, USA). The billet was prepared by Jiangsu Haoran Spray Forming Co., Ltd. (Zhenjiang, China).

**Figure 11:**
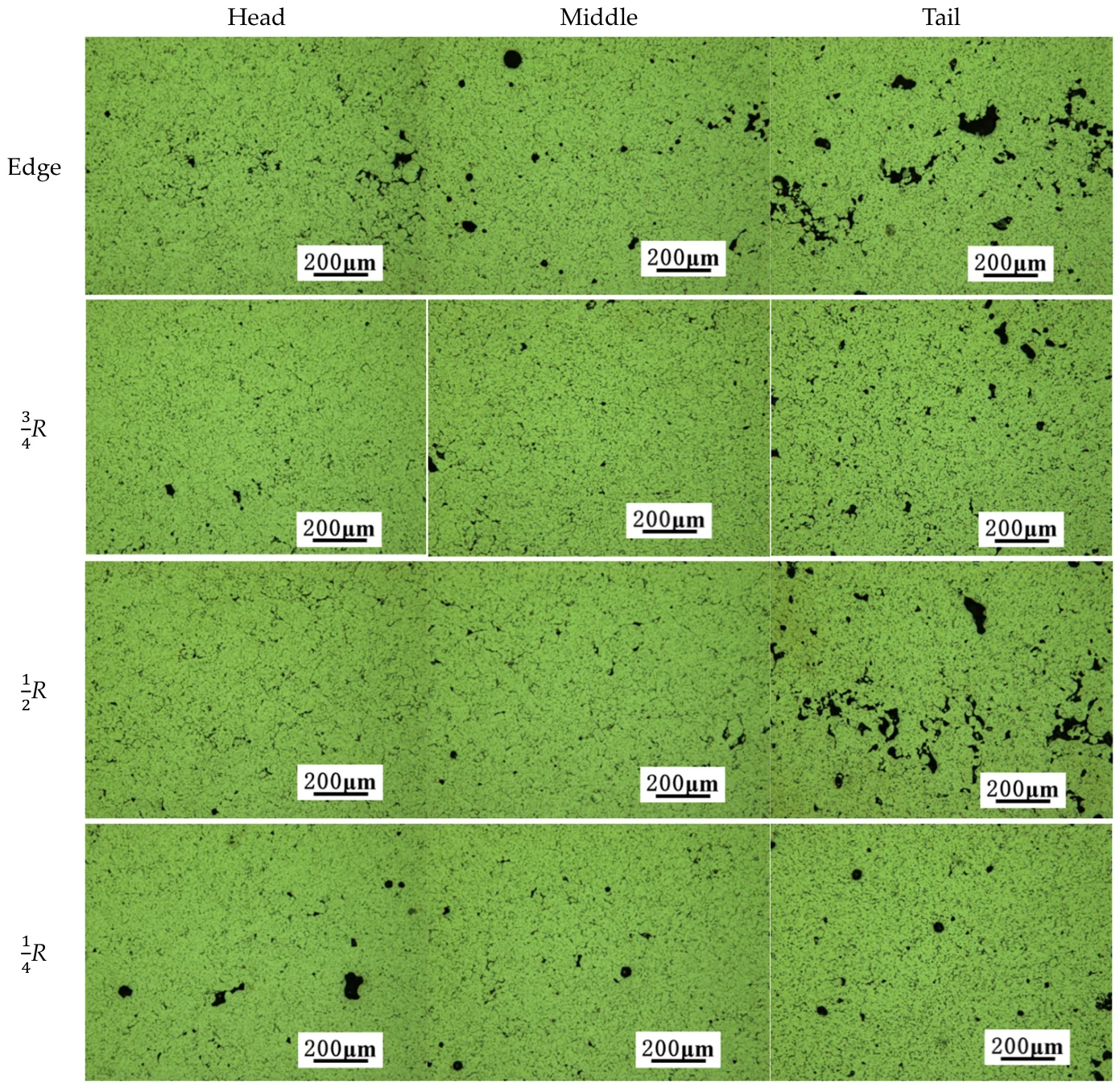

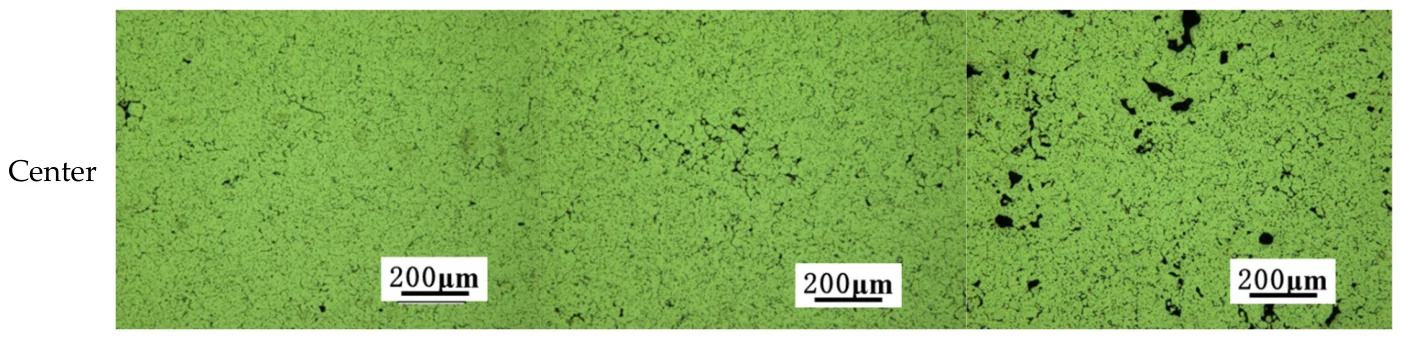
The metallographic structure of billet under the 3−4 arrangement.

The SEM images of the pores in the different parts of the billet are shown in Figure 11. In the head region, the average porosity is approximately 2.3% and the median pore diameter is 2.9 µm. Pores with diameters smaller than 5 µm account for 80% of the total number of pores, and the pore number density is approximately 1170 mm^−2^, indicating a relatively high density of pores. In the middle region, the porosity is approximately 2.6% and the median pore diameter is 2.8 µm. Small pores again predominate, accounting for more than 80% of the total number of pores, and the pore number density is approximately 1385 mm^−2^. The pore distribution is similar to that in the head region, although the defects increase slightly. In the tail region, the number of pores increases markedly and the porosity reaches 7.26%. Although the median pore diameter remains 2.9 µm, both the number of large pores and the maximum pore size increase, and the pore number density rises to 2687 mm^−2^. In the head and tail regions, the microstructure varies considerably along the radial direction. The porosity decreases gradually from the edge toward the center; the porosity at the edge is approximately 4.5 times that at the center, and the number of pores and the pore number density are approximately 3 times those at the center. The tail region shows little variation in the radial direction, with the porosity remaining relatively high at all radial positions. The distribution characteristics of porosity in the billet are attributable primarily to two factors: the excessively large incidence angle at the outer periphery and the bottom, and the deviation of the deposited layer and droplet temperatures from the optimal window around the 50% solid-fraction temperature. In the middle and upper parts, by contrast, the heat input from subsequent deposition gives rise to a self-annealing effect together with adequate feeding, so that the highest relative density is attained there. This pattern is governed mainly by the thermal history and the geometric incidence angle, and is insensitive to the individual process parameters and the billet diameter.

## 4. Conclusions

This study employed ANSYS software to establish a shape model for billets and analyzed the morphology of billets under different atomizer arrangements. The following conclusions were drawn:

(1) With four atomizers positioned at eccentric distances of 141, 245, 316, and 375 mm, respectively, a incidence angle of 45°, scanning angles of 8°, 5°, 3°, and 1°, and a drawing speed of 0.1 mm/s, a large billet with a diameter of 800 mm can be produced.
(2) The pore distribution in the billet exhibited a trend characterized by a higher concentration at the periphery and a lower concentration at the center, as well as a greater prevalence during the initial stage and a reduced occurrence during the middle and later stages.

## Figures and Tables

**Table 1:** Atomizer arrangement.

Process Parameters	Atomizer No. 1	Atomizer No. 2	Atomizer No. 3	Atomizer No. 4
Eccentric Distance L, mm	1-1: L^1^_11_ = 50;	1-1: L^1^_12_ = 71;	1-1: L^1^_13_ = 87;	1-1: L^1^_14_ = 100;
	1-2: L^1^_21_ = 50;	1-2: L^1^_22_ = 80;	1-2: L^1^_23_ = 100;	1-2: L^1^_24_ = 120;
	1-3: L^1^_31_ = 60	1-3: L^1^_32_ = 92	1-3: L^1^_33_ = 113	1-3: L^1^_34_ = 130
Incidence Angle Φ,°	1-1: Φ^1^_11_ = 15;	1-1: Φ^1^_12_ = 15;	1-1: Φ^1^_13_ = 15;	1-1: Φ^1^_14_ = 45;
	1-2: Φ^1^_21_ = 15;	1-2: Φ^1^_22_ = 15;	1-2: Φ^1^_23_ = 15;	1-2: Φ^1^_24_ = 45;
	1-3: Φ^1^_31_ = 15	1-3: Φ^1^_32_ = 15	1-3: Φ^1^_33_ = 15	1-3: Φ^1^_34_ = 45
Scanning Angle α,°	1-1: α^1^_11_ = 5;	1-1: α^1^_12_ = 3;	1-1: α^1^_13_ = 1;	1-1: α^1^_14_ = 0;
	1-2: α^1^_21_ = 5;	1-2: α^1^_22_ = 3;	1-2: α^1^_23_ = 1;	1-2: α^1^_24_ = 0;
	1-3: α^1^_31_ = 5	1-3: α^1^_32_ = 3	1-3: α^1^_33_ = 1	1-3: α^1^_34_ = 0
Atomization Time *T*, s	400	400	400	400
Drawing Speed V, mm/s	1			
Eccentric Distance L, mm	2-1: L^0.5^_11_ = 55;	2-1: L^0.5^_12_ = 100;	2-1: L^0.5^_13_ = 140;	2-1: L^0.5^_14_ = 170;
	2-2: L^0.5^_21_ = 63;	2-2: L^0.5^_22_ = 120;	2-2: L^0.5^_23_ = 150;	2-2: L^0.5^_24_ = 170;
	2-3: L^0.5^_31_ = 65	2-3: L^0.5^_32_ = 120	2-3: L^0.5^_33_ = 150	2-3: L^0.5^_34_ = 170
Incidence Angle Φ,°	2-1: Φ^0.5^_11_ = 15;	2-1: Φ^0.5^_12_ = 15;	2-1: Φ^0.5^_13_ = 15;	2-1: Φ^0.5^_14_ = 45;
	2-2: Φ^0.5^_21_ = 15;	2-2: Φ^0.5^_22_ = 15;	2-2: Φ^0.5^_23_ = 15;	2-2: Φ^0.5^_24_ = 45;
	2-3: Φ^0.5^_31_ = 15	2-3: Φ^0.5^_32_ = 15	2-3: Φ^0.5^_33_ = 15	2-3: Φ^0.5^_34_ = 45
Scanning Angle α,°	2-1: α^0.5^_11_ = 5;	2-1: α^0.5^_12_ = 4;	2-1: α^0.5^_13_ = 3;	2-1: α^0.5^_14_ = 1;
	2-2: α^0.5^_21_ = 5;	2-2: α^0.5^_22_ = 4;	2-2: α^0.5^_23_ = 3;	2-2: α^0.5^_24_ = 1;
	2-3: α^0.5^_31_ = 5	2-3: α^0.5^_32_ = 5	2-3: α^0.5^_33_ = 4	2-3: α^0.5^_34_ = 1
Atomization Time *T*, s	800	800	800	800
Drawing Speed V, mm/s	0.5			
Eccentric Distance L, mm	3-1: L^0.25^_11_ = 75;	3-1: L^0.25^_12_ = 140;	3-1: L^0.25^_13_ = 190;	3-1: L^0.25^_14_ = 240;
	3-2: L^0.25^_21_ = 70;	3-2: L^0.25^_22_ = 170;	3-2: L^0.25^_23_ = 210;	3-2: L^0.25^_24_ = 245;
	3-3: L^0.25^_31_ = 80;	3-3: L^0.25^_32_ = 175;	3-3: L^0.25^_33_ = 205;	3-3: L^0.25^_34_ = 240;
	3-4: L^0.25^_41_ = 90	3-4: L^0.25^_42_ = 180	3-4: L^0.25^_43_ = 210	3-4: L^0.25^_44_ = 250
Incidence Angle Φ,°	3-1: Φ^0.25^_11_ = 15;	3-1: Φ^0.25^_12_ = 15;	3-1: Φ^0.25^_13_ = 15;	3-1: Φ^0.25^_14_ = 45;
	3-2: Φ^0.25^_21_ = 15;	3-2: Φ^0.25^_22_ = 15;	3-2: Φ^0.25^_23_ = 15;	3-2: Φ^0.25^_24_ = 45;
	3-3: Φ^0.25^_31_ = 45;	3-3: Φ^0.25^_32_ = 45;	3-3: ^0.25^_33_ = 45;	3-3: Φ^0.25^_34_ = 45;
	3-4: Φ^0.25^_41_ = 45	3-4: Φ^0.25^_42_ = 45	3-4: Φ^0.25^_43_ = 45	3-4: Φ^0.25^_44_ = 45
Scanning Angle α,°	3-1: α^0.25^_11_ = 5;	3-1: α^0.25^_12_ = 5;	3-1: α^0.25^_13_ = 4;	3-1: α^0.25^_14_ = 1;
	3-2: α^0.25^_21_ = 5;	3-2: α^0.25^_22_ = 5;	3-2: α^0.25^_23_ = 4;	3-2: α^0.25^_24_ = 1;
	3-3: α^0.25^_31_ = 5;	3-3: α^0.25^_32_ = 5;	3-3: α^0.25^_33_ = 5;	3-3: α^0.25^_34_ = 1;
	3-4: α^0.25^_31_ = 5	3-4: α^0.25^_32_ = 5	3-4: α^0.25^_33_ = 5	3-4: α^0.25^_34_ = 1
Atomization Time *T*, s	1600	1600	1600	1600
Drawing Speed V, mm/s	0.25			
Eccentric Distance L, mm	4-1: L^0.1^_11_ = 130;	4-1: L^0.1^_12_ = 270;	4-1: L^0.1^_13_ = 330;	4-1: L^0.1^_14_ = 400;
	4-2: L^0.1^_21_ = 150;	4-2: L^0.1^_22_ = 220;	4-2: L^0.1^_23_ = 320;	4-2: L^0.1^_24_ = 390;
	4-3: L^0.1^_31_ = 140	4-3: L^0.1^_32_ = 245	4-3: L^0.1^_33_ = 315	4-3: L^0.1^_34_ = 375
Incidence Angle Φ,°	4-1: Φ^0.1^_11_ = 45;	4-1: Φ^0.1^_12_ = 45;	4-1: Φ^0.1^_13_ = 45;	4-1: Φ^0.1^_14_ = 45;
	4-2: Φ^0.1^_21_ = 45;	4-2: Φ^0.1^_22_ = 45;	4-2: Φ^0.1^_23_ = 45;	4-2: Φ^0.1^_24_ = 45;
	4-3: Φ^0.1^_31_ = 45	4-3: Φ^0.1^_32_ = 45	4-3: Φ^0.1^_33_ = 45	4-3: Φ^0.1^_34_ = 45
Scanning Angle α,°	4-1: α^0.1^_11_ = 5;	4-1: α^0.1^_12_ = 5;	4-1: α^0.1^_13_ = 5;	4-1: α^0.1^_14_ = 3;
	4-2: α^0.1^_21_ = 12;	4-2: α^0.1^_22_ = 10;	4-2: α^0.1^_23_ = 5;	4-2: α^0.1^_24_ = 5;
	4-3: α^0.1^_31_ = 8	4-3: α^0.1^_32_ = 5	4-3: α^0.1^_33_ = 3	4-3: α^0.1^_34_ = 1
Atomization Time *T*, s	8000	8000	8000	8000
Drawing Speed V, mm/s	0.1			

## Data Availability

The original contributions presented in this study are included in the article. Further inquiries can be directed to the corresponding author.

## References

[1] Huang R., Li M., Yang H., Lu S., Zuo H., Zheng S., Duan Y., Yuan X. (2022). Effects of Mg contents on microstructures and second phases of as-cast Al–Zn–Mg–Cu alloys. J. Mater. Res. Technol..

[2] Fan Z., Huang Z., Tao X., Li Q., Pan Y., Tan Y., Lin W., Liu W., Wu H. (2025). One-step Zn-based composites fabrication via double glow plasma for synergistic mechanical-corrosion control and interface mechanism elucidation. J. Mater. Res. Technol..

[3] Lin X., Cao L., Wu X., Tang S., Zou Y. (2022). Precipitation behavior of spray-formed aluminum alloy 7055 during high temperature aging. Mater. Charact..

[4] Grant P.S. (1995). Spray forming. Prog. Mater. Sci..

[5] Jia Y., Cao F., Ning Z., Guo S., Ma P., Sun J. (2012). Influence of second phases on mechanical properties of spray-deposited Al–Zn–Mg–Cu alloy. Mater. Des..

[6] Fan C.-h., Li J., Yang S.-j., Hu Z.-y., Wu Q., Ou L., Wu S. (2025). Effect of rapid cold stamping on microstructure and mechanical properties of spray-formed Al-Zn-Mg-Cu alloy. Trans. Nonferrous Met. Soc. China (Engl. Ed.).

[7] Underhill R.P., Grant P.S., Cantor B. (1993). Microstructure of spray-formed Al alloy 2618. Mater. Des..

[8] Liu M., Cui Z., Sui D. (2018). Microstructure and porosity formation of spray-formed GCr15 steel billets. Procedia Manuf..

[9] Zhang Z., Liu R., Zhou Y., Zhou G., Li D., Zhao Z., Peng Y., Huang D. (2025). Investigation on microstructural evolution of spray formed Al-Zn-Mg-Cu alloy during hot extrusion processes and subsequent solution treatment. J. Alloys Compd..

[10] Deng Z., Liu C., Zeng G., Deng Y., Shuai S., Han B., Jiao C., Xu Y., Zhang Y. (2026). Microstructure evolution and defect characteristics in spray-formed Al-Zn-Mg-Cu alloy. J. Alloys Compd..

[11] Mazzer E.M., Afonso C.R.M., Galano M., Kiminami C.S., Bolfarini C. (2013). Microstructure evolution and mechanical properties of Al–Zn–Mg–Cu alloy reprocessed by spray-forming and heat treated at peak aged condition. J. Alloys Compd..

[12] Bai P., Hou X., Zhang X., Zhao C., Xing Y. (2009). Microstructure and mechanical properties of a large billet of spray formed Al–Zn–Mg–Cu alloy with high Zn content. Mater. Sci. Eng. A.

[13] Markus S., Cui C., Fritsching U. (2004). Analysis of deposit growth in spray forming with multiple atomizers. Mater. Sci. Eng. A.

[14] Li Z., Zhang G., Li Z., Zhang Y., Xu W. (2008). Simulation of Gas Flow Field in Laval Nozzle and Straight Nozzle for Powder Metallurgy and Spray Forming. J. Iron Steel Res. Int..

[15] Hattel J.H., Pryds N.H. (2006). Modelling the influence of the gas to melt ratio on the solid fraction of the surface in spray formed billets. Simul. Model Pract. Theory.

[16] Zhang G., Li Z., Zhang Y., Mi J., Grant P.S. (2010). Communication: Modeling the deposition dynamics of a twin-atomizer spray forming system. Metall. Mater. Trans. B.

[17] Cui C., Schulz A. (2013). Modeling and simulation of spray forming of clad deposits with graded interface using two scanning gas atomizers. Metall. Mater. Trans. B.

[18] Mi J., Grant P.S., Fritsching U., Belkessam O., Garmendia I., Landaberea A. (2008). Multiphysics modelling of the spray forming process. Mater. Sci. Eng. A.

[19] Pryds N.H., Hattel J.H. (2005). Spray forming: A numerical investigation of the influence of the gas to melt ratio on the billet surface temperature. Int. J. Therm. Sci..

[20] Chourasiya S.K., Gautam G., Kumar A. (2021). Spray forming technique for aluminium matrix materials: A review. Mater. Today Proc..

[21] Lin X., Wu X., Cao L., Bai M., Meng Y. (2025). Optimization of flow stress model and 3D-processing map for spray-formed aluminum alloy 7055 based on GA-BP artificial neural network. J. Alloys Compd..

[22] Kang S., Chang D.-H. (1999). Modelling of billet shapes in spray forming using a scanning atomizer. Mater. Sci. Eng. A.

[23] Li P., Wei S., Lei X., Yang L., Sun B. (2023). Study on Flight Dynamics and Heat Transfer Solidification of Metal Droplets during Centrifugal Spray Deposition Forming Process. Metals.

[24] Liu B., Xu K.-L., Zhang Y.-Y., Ge J. (2022). Gas-liquid dual phase inhibition method for explosion accident of wet Al dust collection system based on KH2PO4. Adv. Powder Technol..

[25] Hussain S., Buss L., Yao D., Fritsching U., Uhlenwinkel V. (2022). Droplet velocity and thermal state from hot gas atomization of steel melt: Impact on the quality of the spray-formed tubular deposit. Adv. Powder Technol..

[26] Cui C., Fritsching U., Schulz A., Li Q. (2005). Mathematical modeling of spray forming process of tubular preforms: Part 1. Shape evolution. Acta Mater..

[27] Djurić Z., Grant P. (2003). An inverse problem in modelling liquid metal spraying. Appl. Math. Model.

[28] Hattel J.H., Pryds N.H. (2004). A unified spray forming model for the prediction of billet shape geometry. Acta Mater..

[29] Gutierrez-Miravete E., Lavernia E.J., Trapaga G.M., Szekely J., Grant N.J. (1989). A Mathematical Model of the Spray Deposition Process. Metall. Trans. A.

[30] Yin Y., Zhang Y., Xiong B., Wang F., Liu H., Li Z., Li X. (2014). Distribution and Evolution of Porous Defects in Spray Formed 7XXX Aluminum Alloy. Rare Metal. Mater. Eng..

[31] Zhang J.S., Cui H., Duan X.J., Sun Z.Q., Chen G.L. (2000). An analytical simulation of solidification behavior within deposited preform during spray forming process. Mater. Sci. Eng. A.

[32] Zhang J.G., Shi H.S., Sun D.S. (2003). Research in spray forming technology and its applications in metallurgy. J. Mater. Process Technol..

